# Development and Characterization of Monoclonal Antibodies to Yellow Fever Virus and Application in Antigen Detection and IgM Capture Enzyme-Linked Immunosorbent Assay

**DOI:** 10.1128/CVI.00209-16

**Published:** 2016-08-05

**Authors:** Ferdinard Adungo, Fuxun Yu, David Kamau, Shingo Inoue, Daisuke Hayasaka, Guillermo Posadas-Herrera, Rosemary Sang, Matilu Mwau, Kouichi Morita

**Affiliations:** a Department of Virology, Institute of Tropical Medicine, Nagasaki University, Nagasaki, Japan; b Graduate School of Biomedical Sciences, Nagasaki University, Nagasaki, Japan; c Kenya Medical Research Institute, Nairobi, Kenya; University of South Carolina School of Medicine Greenville

## Abstract

Yellow fever (YF) is an acute hemorrhagic viral infection transmitted by mosquitoes in Africa and South America. The major challenge in YF disease detection and confirmation of outbreaks in Africa is the limited availability of reference laboratories and the persistent lack of access to diagnostic tests. We used wild-type YF virus sequences to generate recombinant envelope protein in an Escherichia coli expression system. Both the recombinant protein and sucrose gradient-purified YF vaccine virus 17D (YF-17D) were used to immunize BALB/c mice to generate monoclonal antibodies (MAbs). Eight MAbs were established and systematically characterized by indirect enzyme-linked immunosorbent assay (ELISA), Western blot analysis, and immunofluorescence assay (IFA). The established MAbs showed strong reactivity with wild-type YF virus and recombinant protein with no detectable cross-reactivity to dengue virus or Japanese encephalitis virus. Epitope mapping showed strong binding of three MAbs to amino acid positions 1 to 51, while two MAbs mapped to amino acid positions 52 to 135 of the envelope protein. The remaining three MAbs did not show reactivity to envelope fragments. The established MAbs exert no neutralization against wild-type YF and 17D viruses (titer of <10 for both strains). The applicability of MAbs 8H3 and 3F4 was further evaluated using IgM capture ELISA. A total of 49 serum samples were analyzed, among which 12 positive patient and vaccinee samples were correctly identified. Using serum samples that were 2-fold serially diluted, the IgM capture ELISA was able to detect all YF-positive samples. Furthermore, MAb-based antigen detection ELISA enabled the detection of virus in culture supernatants containing titers of about 1,000 focus-forming units.

## INTRODUCTION

Yellow fever virus (YFV) is the prototype virus of the family Flaviviridae, genus Flavivirus. It is an enveloped single-stranded, positive-sense RNA virus transmitted to humans by bites of infected mosquitoes (1). It is closely related to dengue virus (DENV), Japanese encephalitis virus (JEV), and West Nile virus (WNV) (2). The virus RNA encodes three structural proteins, the capsid (C), premembrane/membrane (prM/M), and envelope (E) proteins, and seven nonstructural (NS) proteins, NS1, NS2A, NS2B, NS3, NS4A, NS4B, and NS5.

Clinical symptoms of yellow fever (YF) range from mild febrile disease to severe forms with hemorrhagic manifestations, hepatitis, jaundice, renal failure, rapid terminal events with shock, and multiorgan failure, with case fatality rates exceeding 20%. To date, YF remains a major public health concern for 34 countries in Africa and 14 countries in South America, with a combined population of over 900 million people estimated to be at risk of infection (3).

Although YF is a disease that is preventable by the use of vaccine, 1.7 million cases of YF are estimated to occur annually in Africa, resulting in 29,000 to 60,000 deaths (4, 5). YF outbreaks have been reported in East African countries such as Kenya (1992 to 1993), Uganda (2010 to 2011), and Sudan (2003, 2005, and 2012) (6–11). Considerably larger outbreaks have been reported in West African countries such as Ghana (1977 to 1983), Nigeria (1986 to 1994), and Guinea (2000 to 2005) (12, 13). Over the past 10 years, many countries in which yellow fever is endemic such as countries in West Africa and some in Central Africa such as Central African Republic, Congo, and Chad have reported YF cases to the World Health Organization (WHO) (14, 15).

Clinical diagnosis of YF is difficult due to the common occurrence of other, symptomatically similar endemic diseases such as malaria, typhoid fever, viral hepatitis, dysentery, and other viral hemorrhagic fevers. Laboratory diagnosis of YF faces several challenges, such as a lack of commercial test kits, a lack of biosafety level 3 (BSL3) laboratories for virus isolation, and the presence of serological cross-reactivity with other flavivirus infections. A number of molecular diagnostic methods such as reverse transcription-PCR (RT-PCR) (16), real-time RT-PCR (17, 18), real-time RT–loop-mediated isothermal amplification (RT-LAMP) (19, 20), and real-time RT-RNase protection assay (RT-RPA) (21) have been developed for laboratory diagnosis of YF. Although these techniques are rapid, highly sensitive, and accurate, the cost of equipment and reagents limits their use. In addition, virus isolation and cell culture systems require BSL3 laboratories.

A number of antibody (Ab)-based diagnostic tests have been developed for detection of dengue virus (DENV), West Nile virus (WNV), and Japanese encephalitis virus (JEV) using monoclonal and polyclonal antibodies (22–27). However, not much has been reported on the development of such diagnostic tests for YFV (28). The use of both monoclonal and polyclonal antibodies in the development of diagnostic tests for YF could help to address the lack of commercial kits and improve access to testing outside regional or reference laboratories that currently serve countries where YF is endemic. Here we report the development, characterization, and evaluation of YF monoclonal antibodies (MAbs). Diagnostic application of these MAbs was tested using antigen detection and IgM capture enzyme-linked immunosorbent assays (ELISA).

## MATERIALS AND METHODS

### Ethics statement.

The Institutional Animal Care and Use Committee (IACUC) of Nagasaki University approved the mouse experimental procedures. The Kenya Medical Research Institute (KEMRI) Ethical Review Board (SSC no. 1829) granted permission for the use of human samples.

### Serum samples.

Anonymized human serum samples were obtained from KEMRI (reference laboratory for hemorrhagic fever viruses) for validation of IgM capture ELISA. A total of 49 serum samples, comprising 37 negatives and 12 YF IgM positives, were evaluated. The 37 negative serum samples were from 30 healthy volunteers, 4 dengue patients, and 3 Japanese encephalitis patients not exposed to YF as previously determined by laboratory testing. Of the 12 YF IgM positives, 6 were from YF patients infected naturally during the 1992 to 1993 YF outbreak in Kenya (8), while the remaining 6 were from healthy YF vaccine recipients. All 37 of the YF-negative individuals had no history of YF vaccination. The dengue virus and JEV IgM-positive serum samples were included to evaluate the specificity of the IgM capture ELISA.

### Viruses and cells.

YF vaccine virus strain 17D-204, wild-type YF virus (strains Baringo 1 and Baringo 2) isolated from human cases during the 1992–1993 YF outbreak in Kenya (Keiyo district) (8), dengue virus serotype 2 (DENV-2 strain 00St-22A) randomly selected as a representative dengue serotype, and Japanese encephalitis virus (JEV strain JaOArS982) were cultured in Vero cells (African green monkey kidney cell line [ATCC CCL81]). The cells were maintained at 37°C in Eagle's minimum essential medium (MEM) containing 10% fetal calf serum (FCS) and 0.2 mM nonessential amino acids (NEAA). Confluent monolayers of Vero cells were inoculated with the respective viruses, incubated at 37°C, and observed daily for cytopathic effect (CPE). When 20% of the cells showed CPE, the cells were harvested and fixed on slides using cold acetone for immunofluorescence assay (IFA) to determine cross-reactivity of the MAbs to DENV-2 and JEV.

### Virus purification.

Sucrose gradient ultracentrifugation was used to purify YF vaccine virus 17D, DENV-2, and JEV as previously described by Inoue et al. (29). Briefly, the cell culture supernatant was harvested 5 days after inoculation with virus and concentrated using NaCl and polyethylene glycol (PEG) 6000 (Wako Chemicals, Tokyo, Japan). The supernatant was subjected to sucrose gradient ultracentrifugation at 50,000 × *g* for 18 h at 4°C. The presence of virus was confirmed using indirect IgG ELISA.

### Construction of recombinant plasmids.

The wild-type YF virus (strain Baringo 1) was used to inoculate a confluent monolayer of Vero cells at 37°C in MEM supplemented with 10% fetal calf serum (FCS) and 0.2 mM NEAA, and the culture was incubated for 5 days. RNA was extracted from cell supernatants using a QIAamp viral RNA minikit (Qiagen, Hilden, Germany) following the manufacturer's instructions. The amplification was done using Superscript III One-Step RT-PCR mix (Invitrogen, Carlsbad, CA, USA) with YF virus-specific primers designated YFV-Ep1 (sense; 5′-TCAGGATCCTGCATTGGAATTACT-3′) and YFV-Ep2 (antisense; 5′-CAACAAGCTTATTGAGCTTCCCT-3′) to generate a truncated envelope gene of YF virus. The sense and antisense primers contained recognition sites for BamHI and HindIII (underlined nucleotides), respectively. The positions of the sense and antisense primers correspond, respectively, to nucleotides 978 to 994 and nucleotides 2160 to 2148 of the YF virus genome strain (GenBank accession no. NC_002031) (30). The DNA fragments were digested with the restriction enzymes mentioned above, purified by the use of a Qiaex II gel extraction kit (Qiagen, Hilden, Germany), and subsequently cloned into the corresponding restriction sites of the pQE-30 plasmid vector (Qiagen, Hilden, Germany). The insertion of the recombinant plasmid was confirmed to be in frame by nucleotide sequencing. An expression construct encompassing amino acid positions 3 to 403 of YFV-E protein with a vector-derived His tag (histidine hexamer tag) at the N terminus was obtained. The resultant recombinant plasmid was designated pQE-30-YFV-E.

### Expression, purification, and refolding of recombinant YFV-E protein.

The recombinant YFV-E protein was expressed by transforming recombinant plasmid pQE-30-YFV-E into Escherichia coli strain XL-1 Blue. The E. coli strain was cultured at 37°C in Luria-Bertani (LB) medium supplemented with ampicillin (100 μg/ml). The expression of recombinant proteins was induced by the addition of 0.5 mM isopropyl β-d-thiogalactoside (IPTG; Invitrogen, CA, USA) for 3 h. The cells were centrifuged at 18,600 × *g* for 30 min at 4°C. The cell pellet was resuspended in 30 ml phosphate-buffered saline (PBS) (pH 7.5) and then sonicated for 5 min on ice. The inclusion body (IB) pellet was suspended in 100 ml phosphate-buffered saline (PBS) (pH 8.0) containing 50 mM Tris-HCl, 1 mM EDTA, and 2% Triton X-100 followed by incubation at room temperature for 10 min and was then centrifuged at 20,000 × *g* at 4°C for 20 min. The purified IB pellet was solubilized with IB solubilization buffer (pH 8.0) containing 8 M urea, 20 mM sodium phosphate, 500 mM NaCl, and 1 mM β-mercaptoethanol and stirred for 1 h at room temperature. The suspension was centrifuged at 20,000 × *g* at 4°C for 30 min, and the supernatant was collected for further purification. The recombinant protein was purified by immobilized metal affinity chromatography using a nickel-nitrilotriacetic acid (Ni-NTA) resin column (Qiagen, Hilden, Germany) by following the manufacturer's instructions. The purity of eluted protein was analyzed by using sodium dodecyl sulfate-polyacrylamide gel electrophoresis (SDS-PAGE). Protein refolding was done by slow dilution of the urea-denatured proteins in 2 liters of refolding buffer overnight. The refolding buffer (pH 8.0) contained 50 mM Tris-HCl, 0.4 M l-arginine (Gibco, NY, USA), 1.0 mM GSH (glutathione [reduced form]), and 0.1 mM GSSH (glutathione [oxidized form]) (Sigma, St. Louis, MO, USA). The refolded protein was further dialyzed in PBS (pH 7.2) at 4°C for 48 h with a change of buffer every 12 h. Protein concentrations were determined by using a Bio-Rad protein assay reagent kit (Bio-Rad, CA, USA), and the protein was stored at −20°C until use.

### Production of YFV MAbs.

Using the prime-boost approach, two groups of three 6- to 8-week-old female BALB/c mice were immunized intraperitoneally with either recombinant YFV-E protein or sucrose gradient-purified YF vaccine virus 17D mixed with equal volumes of Freund's complete adjuvant (MP Biochemicals, CA, USA) at a dose of 100 μg per injection. Two boosts of the same antigen mixed with Freund's incomplete adjuvant (MP Biochemicals) were given at 2-week intervals. Mice were bled 7 days after the second booster immunization, and indirect ELISA was performed to check antibody titers. Finally, 100 μg of the antigen without adjuvant was administered for three consecutive days prior to harvesting spleen cells for fusion with SP2/0 myeloma cells performed using PEG 1500 (Roche, Indianapolis, IN, USA). The hybridoma cells were grown in selective medium (hypoxanthine-aminopterin-thymidine [HAT]; Gibco, NY, USA) for 10 days before screening was performed by indirect ELISA to select cells producing antibodies against YFV-E protein and YF vaccine virus 17D. Positive clones were further subjected to limiting dilution to establish single stable clones. Briefly, a hybridoma cell suspension was diluted in growth medium (RPMI 1640; Gibco, NY, USA) supplemented with 10% FCS and seeded onto 96-well microplates to about 1 cell per well. After 10 days of incubation, indirect ELISA was performed to select clones secreting desired antibodies. Positive clones were transferred to culture flasks for propagation using growth media. MAb isotypes were determined using a mouse MAb isotyping kit (Pierce Biotechnology, Rockford, IL, USA) according to the manufacturer's instructions. Large-scale propagation of positive clones was done using Hybridoma-SFM medium (Gibco, NY, USA) followed by MAb purification using MAb HiTrap protein G chromatography (GE Healthcare, Uppsala, Sweden).

### Screening of MAbs by indirect ELISA.

Hybridoma cell supernatants were screened for the presence of antibody using indirect IgG ELISA. To ensure the selection of all positive clones, the screening ELISA utilized the same antigen used for immunization of the mice. Briefly, a 96-well microplate (Nunc; Maxisorp, Denmark) was coated with either purified YFV-E at 100 ng per well or sucrose gradient-purified YF vaccine virus 17D at 250 ng per well in ELISA coating buffer (0.01 M PBS; pH 7.4) at 4°C overnight. To avoid nonspecific binding, the wells were blocked with 100 μl of the original concentration of BlockAce (Yukijirushi, Sapporo, Japan) and incubated for 1 h at room temperature. The microplate was washed three times with PBS containing 0.05% Tween 20 (PBS-T) (Gibco, NY, USA). A 100-μl volume of hybridoma culture supernatant was added to each well, and the mixture was incubated for 1 h at 37°C. Preimmunization and postimmunization mouse serum samples were used for negative and positive controls, respectively. The microplate was washed three times with PBS-T, 100 μl of goat anti-mouse IgG conjugated to horseradish peroxidase (HRP) (American Qualex, San Clemente, CA) at a 1:10,000 dilution in BlockAce was added per well, and the mixture was incubated for 1 h at 37°C. The microplate was again washed three times with PBS-T, and 100 μl of ABTS substrate [2,2′ azinobis (3-ethylbenzthiazolinesulfonic acid)] solution (Roche Diagnostics, Mannheim, Germany) was added to each well. The microplate was incubated in the dark at room temperature for 30 min, and the optical density at 405 nm (OD_405_) was measured using a Multiskan ELISA plate reader (Thermolabsystem, Tokyo, Japan). All clones with an optical density two times or higher that of the negative control were considered positive.

### SDS-PAGE and Western blot analysis.

The YFV-E protein was analyzed by SDS-gel electrophoresis in a 5% to 20% polyacrylamide gel (ATTO, Japan). Briefly, 5 μl of protein (2 mg/ml) was mixed with 5 μl of loading buffer (pH 7.4) containing 1% SDS, 25 mM Tris-HCl, 0.5% β-mercaptoethanol, and 0.001% bromophenol blue and then incubated at 95°C for 5 min before loading onto the gel was performed. Staining was done using Coomassie blue G250 reagent (Bio-Rad, USA) for 1 h, and the reaction mixture was destained in distilled water overnight. Western blot analysis was done following the protocol of Yu et al. (31). Hybridoma clones that were not reactive in Western blotting were excluded from further characterization.

### Immunofluorescence test.

To determine the reactivity of MAbs with flaviviruses, Vero cells infected with YFV (strains Baringo 1, Baringo 2, and 17D), JEV, and DENV-2 were harvested and centrifuged at 600 × *g* for 7 min. The cell pellet was washed three times with PBS and applied to a Teflon-coated eight-multiwell glass slide (MP Biochemicals, CA, USA). After complete drying, cells were fixed for 10 min in cold acetone at 4°C. The slides were blocked using BlockAce and incubated for 30 min at 37°C in a moist chamber. The slides were washed three times in PBS with slow shaking and then air-dried for 10 min at room temperature. A 15-μl volume of MAb cell supernatant diluted at 1:10 in BlockAce was applied to each test well, and the reaction mixture was incubated for 1 h in a moist chamber at 37°C. Anti-flavivirus MAb 12D11/7E8 (32–35) cell supernatant was used as a positive control for DENV and JEV. Commercial anti-YFV MAb 2D12 (Sigma, St. Louis, MO, USA) was used as a positive control for both wild-type virus and 17D vaccine virus. The slides were washed three times in PBS and air-dried for 10 min at room temperature. Next, 15 μl of fluorescein isothiocyanate (FITC), conjugated goat anti-mouse IgG (Bethyl Laboratories Inc., Montgomery, TX, USA) was applied at a dilution of 1:50 to each test well and the mixture was incubated for 1 h at 37°C in the dark. The slides were washed three times in PBS and air-dried for 10 min and finally examined by fluorescence microscopy.

### Indirect ELISA to determine MAb cross-reactivity to other flaviviruses.

To check for cross-reactivity to other flaviviruses, all positive hybridoma clones were analyzed by indirect ELISA using sucrose gradient-purified YF vaccine virus 17D, DENV-2, and JEV as assay antigens. Microplate wells were coated with purified virus in ELISA coating buffer at a concentration of 250 ng per well and incubated at 4°C overnight. The subsequent steps for blocking, washing, and incubation were similar to those described above for screening of hybridoma by indirect ELISA. The antibodies used for positive controls were similar to those described earlier for the IFA. Hybridoma growth medium (RPMI 1640) was used as a negative control. After a reaction was performed with 100 μl of HRP-conjugated goat anti-mouse IgG for 1 h at 37°C, the microplate reaction was developed by adding 100 μl of ABTS substrate solution per well. The clones with an OD_405_ value two or more times higher than that of the negative control were considered positive.

### Cloning and expression of YFV-E protein fragments.

X-ray crystallography analysis of tick-borne encephalitis virus (TBEV) and, recently, DENV-2 structures of the E protein ectodomain revealed three distinct domains (domains I, II, and III). The soluble fragment of the DENV-2 E protein ectodomain contains three segments of domain I (residues 1 to 51, 132 to 190, and 278 to 293), two segments of domain II (residues 52 to 131 and 191 to 277), and a single domain III segment (residues 294 to 391) (36–38). In this study, the YFV E protein ectodomain was subdivided into six fragments corresponding to the residues of a soluble fragment of the DENV-2 E protein ectodomain (see Fig. S1 in the supplemental material). The fragments were expressed in E. coli strain XL-1 Blue using maltose-binding protein (MBP) in a soluble form. The genes encoding the protein of interest were PCR amplified using specific primers and cloned into pMAL-c5X plasmid vector (New England BioLabs, Ipswich, United Kingdom). The primers used for PCR, the peptide sequence, and the nucleotide positions of the six fragments are summarized in Table S1 in the supplemental material.

The insertions were confirmed to be in frame by sequencing. The MBP-fusion proteins expressed cytoplasmically were purified by amylose affinity chromatography and dialyzed in PBS (pH 7.2) at 4°C for 24 h with a change of buffer every 12 h. The MBP-fused envelope fragments were analyzed by SDS-PAGE and used for epitope mapping of the MAbs generated in this study.

### Epitope mapping of YFV MAbs by indirect ELISA.

Using indirect IgG ELISA, a 96-well microplate was coated with purified MBP-fusion protein of each fragment in ELISA coating buffer at 100 ng per well and incubated at 4°C overnight. Blocking of the wells was done as mentioned above. After washing three times with PBS-T was performed, hybridoma cell supernatants from all eight of the MAbs (3B6, 3F4, 4A1, 4C9, 4H10, 5B6, 5H2, and 8H3) were applied to each fragment and the reaction mixtures were incubated for 1 h at 37°C. The microplate was again washed three times with PBS-T, 100 μl of HRP-conjugated goat anti-mouse IgG diluted 1:10,000 was applied to the wells, and the reaction mixtures were incubated for 1 h at 37°C. After the final washing step, 100 μl of ABTS substrate solution was added to each well and the reaction mixtures were incubated for 30 min at 37°C in the dark. The mean OD_405_ value for each MAb was calculated from three independent tests.

### Neutralization test.

The neutralization activity of the MAbs was investigated in Vero cells as previously described (35) using wild-type YFV strain Baringo 2 and YF vaccine virus 17D. Briefly, each virus-infected culture fluid (ICF) reaction mixture containing 100 focus-forming units (FFU) was mixed with the diluted MAb to allow the virus-antibody neutralization reaction. After 1 h of incubation at 37°C, 100 μl of the mixture was inoculated onto a Vero cell monolayer and the reaction mixtures were incubated for 90 min at 37°C and then overlaid with MEM containing 2% FCS and 1.25% methylcellulose. After incubation for 96 h at 37°C was performed, focus immunostaining was conducted and the number of foci of infected cells was determined by microscopy. The 50% focus reduction neutralization titer (FRNT_50_) was calculated using the reciprocal of the MAb dilution that reduced the number of foci of infected cells by at least 50% or more in wells with MAb compared to the negative-control wells with no antibody.

### YFV antigen detection ELISA using newly developed MAbs.

The applicability of the MAbs for the development of antigen detection ELISA was evaluated using YF-17D and recombinant YFV-E protein. Several MAb combinations were assessed, and MAb 4C9 (capture antibody) and MAb 3F4 (labeled antibody) were selected based on the results of the previous IgG indirect ELISA. The 96-well microplate was coated with 100 μl of purified MAb 4C9 in ELISA coating buffer at 100 ng per well and incubated at 4°C overnight. The amount of antibody used for coating the wells was determined by checkerboard titration performed with YFV-E protein. Serial dilutions of YF-17D-infected culture fluid (YF-17D ICF) originally containing 3.0 × 10^5^ FFU/100 μl were applied, and the reaction mixtures were incubated for 1 h at 37°C. Similarly, 100 μl of serially diluted (20 μg to 2.0 pg) YFV-E protein was added to each well and the microplate was incubated for 1 h at 37°C. The microplate was washed three times with PBS-T, and 100 μl of in-house HRP-conjugated MAb 3F4 diluted 1:500 in BlockAce was added to all wells. After incubation for 1 h at 37°C, and a final washing step, 100 μl of ABTS substrate solution was applied to each well and the microplate was incubated for 30 min at 37°C. The mean OD_405_ values were measured for each antigen concentration.

### Application of MAbs in YFV IgM capture ELISA.

To evaluate the possibility of using our MAbs for the diagnosis of YF, an IgM capture ELISA for analysis of human serum was developed and evaluated. A total of 49 serum samples were tested. Twelve IgM-positive samples from six YF vaccine recipients (coded Pos1 to Pos6) and six patient samples obtained from the 1992–1993 YF outbreak in Kenya (coded Kd1 to Kd6) were analyzed. In addition, 37 negative serum samples from 30 healthy nonvaccinated volunteers, 4 DENV patient samples, and 3 JEV patient samples were included to evaluate the specificity of the IgM capture ELISA. Briefly, each well of a 96-well microplate was coated with 100 μl of 5.5 μg anti-human IgM (goat IgG) (Cappel ICN Pharmaceuticals, Aurora, OH) dissolved in ELISA coating buffer. The microplate was incubated at 4°C overnight and blocked as mentioned earlier. Human serum samples diluted at 1:200 in 5% skim milk powder (Difco, Detroit, USA)–PBS-T were added and incubated at 37°C for 1 h. After washing three times with PBS-T was performed, 100 μl of YF-17D ICF was applied to each well and the reaction mixture was incubated for 1 h at 37°C. The virus was removed by washing the microplate three times in PBS-T before adding 100 μl of either MAb 3F4 or MAb 8H3. The microplate was incubated for 1 h at 37°C. After washing three times with PBS-T was performed, 100 μl of HRP-conjugated goat anti-mouse IgG (American Qualex, USA) diluted 1:10,000 in BlockAce was applied to each well and the microplate was incubated for 1 h at 37°C. After a final washing step, 100 μl of ABTS substrate solution was added to each well and the microplate was incubated for 30 min at 37°C in a dark chamber. The OD_405_ values were determined from three independent tests. All serum samples with mean OD_405_ values yielding a positive/negative ratio equal to or greater than 2.0 were considered positive for IgM antibodies against YFV. Next, in order to check for the sensitivity of the ELISA, 2-fold serial dilutions of all serum samples positive for IgM antibodies were done starting from 1:200 until a 1:25,600 dilution was obtained. Analysis was done as described above.

## RESULTS

### Expression and purification of recombinant YFV-E protein.

The amplification of the target gene by RT-PCR using specific primers resulted in a 1,200-bp DNA fragment encoding a truncated recombinant YFV-E protein. The recombinant YFV-E protein was purified by affinity chromatography and successfully refolded. SDS-PAGE analysis of purified protein showed a single protein band of 42 kDa as predicted by amino acid sequence analysis.

### Generation of MAbs to YFV.

Fusion of spleen cells from immunized BALB/c mice with SP2/0 myeloma cells generated a total of 26 positive hybridoma clones as determined by indirect ELISA. Fifteen of these clones were from mice immunized with E. coli-expressed YFV-E protein, and the remaining 11 clones were from mice immunized with YF-17D. Following successive subcloning by limiting dilution and confirmation by Western blotting and IFA, a total of 18 hybridoma clones were excluded from further characterization and evaluation due to genetic instability and loss of function. As a result, eight MAbs against YFV were obtained: four MAbs (designated 4A1, 4C9, 5H2, and 4H10) were obtained from mice immunized with recombinant YFV-E protein, and four MAbs (designated 3F4, 8H3, 5B6, and 3B6) were obtained from YF-17D-immunized mice.

We performed heavy-chain analysis of the eight MAbs and revealed four IgG1 subclasses, three IgG2b subclasses, and a single IgG2a subclass. In addition, all of the MAbs had kappa light chains. We further determined the reactivity of the MAbs with denatured YFV-E protein by Western blotting. The eight MAbs reacted with a 42-kDa YFV-E protein band. To assess the cross-reactivity of the MAbs with other flaviviruses, we performed indirect ELISA using sucrose gradient-purified DENV-2, JEV, and YF-17D. The microplate coated with YF-17D showed positive reactivity for all of the clones, while the microplates coated with purified DENV-2 and JEV were negative. These results indicated that the generated MAbs were YF virus specific. We obtained all clones of MAb IgG by HiTrap protein G affinity purification from large-scale cultures in serum-free media.

**FIG 1:**
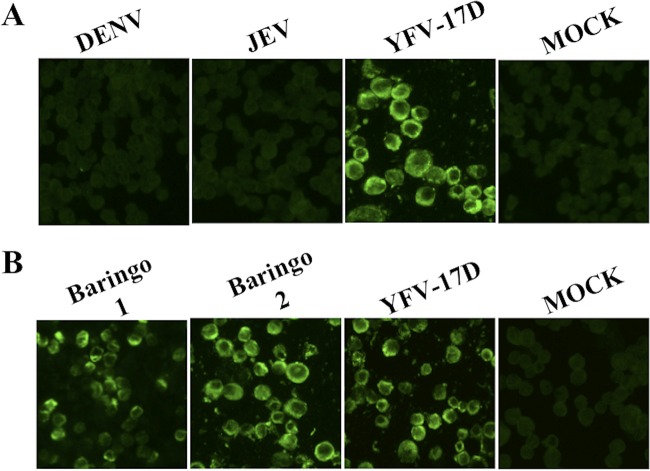
Immunofluorescence analysis of MAb 3F4 to DENV-, JEV-, and YFV-infected Vero cells. (A) MAb 3F4 showed strong fluorescence with YF-17D virus-infected Vero cells but not with DENV-2- and JEV-infected Vero cells. (B) MAb 3F4 showed strong reactivity to wild-type YFV strains Baringo 1, Baringo 2, and 17D. Similarly, all seven of the remaining MAbs showed strong fluorescence with YFV strains and no fluorescence with DENV-2- or JEV-infected Vero cells (images not shown).

Next, we evaluated the specificity of the developed MAbs by IFA using DENV-2-, JEV-, and YF-17D-infected Vero cells. No fluorescence was observed in Vero cells infected with DENV-2 and JEV. As expected, all of the clones showed strong fluorescence with Vero cells infected with YF-17D. An example of IFA of MAb 3F4 with DENV-2- and JEV-infected Vero cells is shown in Fig. 1A. These results confirmed that the eight MAbs were specific for YFV and had no cross-reactivity with DENV-2 and JEV. This was further verified using wild-type YFV strains Baringo 1 and Baringo 2 obtained from a previous YFV outbreak in Kenya. The MAbs showed strong reactivity with wild-type YF viruses as well as with vaccine virus 17D. An example of IFA of MAb 3F4 with YF-17D and with wild-type YF virus strains Baringo 1 and Baringo 2 is shown in Fig. 1B. The properties of the eight MAbs are summarized in Table 1.

**TABLE 1:** Summary of the properties of the established MAbs analyzed by different serological assays

MAb clone	Immunogen	Isotype	Indirect IgG ELISA^*a*^			IFA result^*b*^					YFV-E Western blot result (E. coli)^*c*^	Domain specificity^*d*^	FRNT_50_^*e*^	
			YF-17D	DENV2	JEV	YF-17D	Baringo 1	Baringo 2	DENV2	JEV			YF-17D	Baringo 2
5H2	E protein	IgG2bκ	+	−	−	++	++	++	−	−	++	DoIIR1	<10	<10
4A1	E protein	IgG1κ	+	−	−	+	+	+	−	−	+	ND	<10	<10
4C9	E protein	IgG1κ	+	−	−	++	++	++	−	−	++	DoIR1	<10	<10
4H10	E protein	IgG2bκ	+	−	−	++	++	++	−	−	++	DoIR1	<10	<10
3B6	17D virus	IgG2bκ	+	−	−	+	+	+	−	−	+	ND	<10	<10
5B6	17D virus	IgG1κ	+	−	−	+	+	+	−	−	+	DoIIR1	<10	<10
3F4	17D virus	IgG2aκ	+	−	−	++	++	++	−	−	+	DoIR1	<10	<10
8H3	17D virus	IgG1κ	+	−	−	++	++	++	−	−	+	ND	<10	<10

a The reactivity of MAbs with selected flaviviruses was determined by indirect IgG ELISA. +, positive; −, negative.

b IFA fluorescence intensity was scored as follows: −, no fluorescence detectable; +, intermediate reactivity; ++, high fluorescence intensity.

c The reactivity of MAbs with YFV-E protein was determined by Western blot analysis. −, no detectable signal; +, weak positive signal; ++, strong positive signal.

d ND, not determined.

e FRNT_50_, neutralization titer. The neutralization titer was determined as the reciprocal of the MAb dilution that reduced the number of foci by 50% or more in wells with MAb compared to negative-control wells.

### Epitope mapping of YFV MAbs.

**FIG 2:**
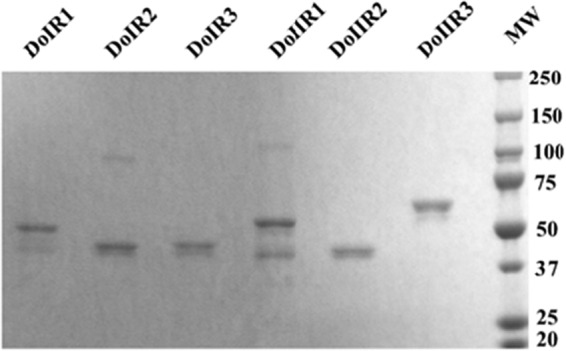
SDS-PAGE analysis of purified YFV-E protein fragments attached to MBP-fusion proteins. YFV-E protein fragments were expressed and purified as MBP-fusion proteins in E. coli. Lanes 1 to 6 indicate individual fragments; MW represents the prestained protein marker.

**FIG 3:**
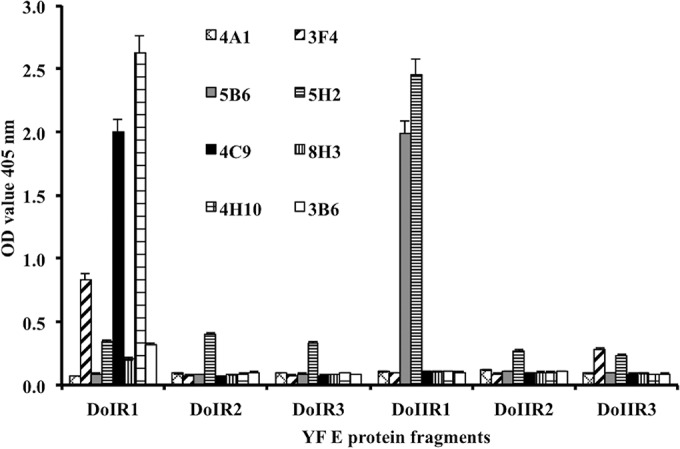
Epitope mapping of YFV MAbs. Indirect ELISA using YFV-E protein fragments as coating antigen was done to identify the epitopes of the generated YFV MAbs. Three MAbs (3F4, 4C9, and 4H10) showed strong reactivity to fragment DoIR1 (amino acids 1 to 51), while two MAbs (5B6 and 5H2) showed strong reactivity to fragment DoIIR1 (amino acids 52 to 135). Three MAbs (3B6, 4A1, and 8H3) did not show strong reactivity to any of the six fragments.

To identify the MAb target epitopes, the six fragments covering the entire coding sequence of the E protein of YFV, designated DoIR1, DoIIR1, Do1R2, DoIIR2, DoIR3, and DoIIR3, were successfully expressed in E. coli in a soluble form and purified by affinity chromatography. The fragments were expressed individually and purified by affinity chromatography. SDS-PAGE analysis of the six fragments revealed the expected protein bands based on the sequence prediction (Fig. 2). The MBP-fused protein fragments indicated molecular weights (MW) of 42 to 60. Smaller fragments showed MW of 42, which indicated the MW of MBP-fusion protein. Of the eight established MAbs, three (3F4, 4C9, and 4H10) showed strong binding to fragment DoIR1 whereas two (5B6 and 5H2) showed strong binding to fragment DoIIR1 of the envelope. The other three MAbs (3B6, 4A1, and 8H3) did not show strong reactivity to any of the six fragments (Fig. 3).

### Virus neutralization activity.

The ability of the established MAbs to neutralize wild-type virus (strain Baringo 2) and YF vaccine virus 17D was evaluated by FRNT_50_ analysis. All of the MAbs showed no neutralization effect against wild-type virus strain Baringo 2 and vaccine virus 17D (neutralization titer, <10). The FRNT_50_ results are summarized in Table 1.

### Antigen detection using newly developed YFV MAbs.

**FIG 4:**
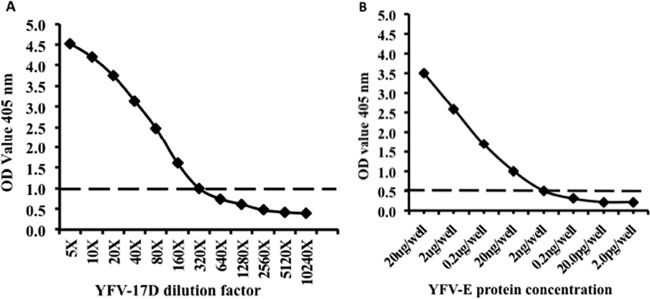
Antigen detection ELISA using newly developed YFV MAbs. (A) Antigen detection using YF vaccine virus 17D-infected culture fluid (YF-17D ICF). The antigen detection ELISA could detect up to 1.0 × 10^3^ FFU/ml of YF-17D virus. (B) The antigen detection ELISA could detect up to 2 ng of YFV-E protein. The dotted line represents the cutoff value (calculated as twice the mean absorbance value of the negative control). Uninfected Vero cell culture supernatants and PBS were used as negative controls for panels A and B, respectively.

Antigen detection ELISA was developed by using the newly generated YFV MAbs. The detection limit of the antigen detection sandwich ELISA was evaluated by using YF-17D ICF and recombinant YFV-E protein. Using MAb 4C9 (capture antibody) and MAb 3F4 (HRP-labeled antibody), we detected YF vaccine virus 17D to a dilution endpoint of 1.0 × 10^3^ FFU/ml (Fig. 4A). On the other hand, this sandwich ELISA was able to detect recombinant YFV-E protein to a dilution endpoint of 2.0 ng/well (Fig. 4B). These results indicated that the antigen detection ELISA is very sensitive and showed the possibility for application in surveillance of YFV in mosquitoes.

### MAb application in IgM capture ELISA.

The IgM capture ELISA performed with either MAb 3F4 or MAb 8H3 as the detecting antibody enabled the correct identification of all 12 YFV IgM-positive samples (6 YF patient samples and 6 vaccinee samples). All of the results of experiments performed with nonvaccine samples were negative. The specificity of the IgM capture ELISA was verified using four known DENV-2 and three JEV IgM-positive samples. All DENV-2 and JEV serum samples were negative, indicating that there was no cross-reactivity with IgM antibody and DENV-2 or JEV.

**FIG 5:**
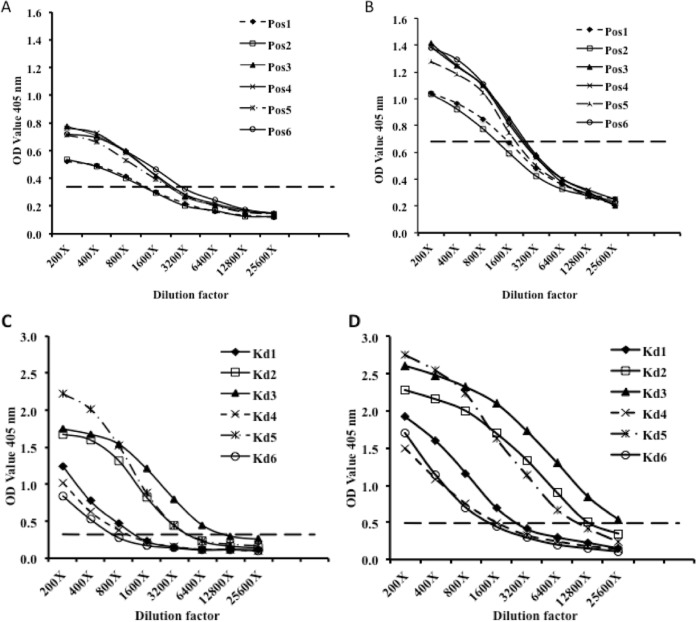
Application of MAbs in IgM capture ELISA. (A and B) Six serially diluted YF vaccinee serum samples (Pos1 to Pos6) were analyzed using MAb 8H3 (A) or MAb 3F4 (B). (C and D) The sensitivity of the IgM capture ELISA was further verified using six serially diluted patient serum samples (Kd1 to Kd6) and analyzed with MAb 8H3 (C) and MAb 3F4 (D).

The sensitivity of the IgM capture ELISA was further verified using serial dilution of all positive samples. For the vaccinee serum samples, both MAb 8H3 and MAb 3F4 showed positive results up to a dilution of 1:800 (Fig. 5A and B, respectively). On the other hand, some serially diluted patient samples showed even higher positive dilution endpoints. Three samples, Kd1, Kd4, and Kd6, were positive at a dilution of 1:800 whereas the other three samples, Kd2, Kd3, and Kd5, were positive at dilutions up to 1:3,200 for both MAb 8H3 and 3F4 (Fig. 5C and D, respectively). Overall, the IgM capture ELISA detected all positive serum samples at a dilution of 1:800. Serum sample Kd3 still showed positive results at a dilution of 1:6,400.

## DISCUSSION

Yellow fever is an acute viral infection transmitted through mosquito bites. It is strongly believed that the current emergence and reemergence of YFV and other arboviruses is partly attributable to the increased migration of people from countries where such diseases are endemic and the expanding establishment of the vector. One of the major challenges in YF laboratory diagnosis is the lack of availability of commercial diagnostic kits. The use of flavivirus antigens in the development of MAbs and diagnostic tests has been documented with very little focus on YF (22, 23, 25–28, 39–42). The application of MAbs in immunoassays offers the advantage of high specificity due to their ability to bind to specific epitopes of the antigen. This is useful in reducing the incidence of cross-reactivity reported to negatively impact the use of many serological kits (43, 44).

In this study, eight monoclonal antibodies with strong and specific reactivity to YFV were developed, systematically characterized, and evaluated for their applicability in the development of YF diagnostic tests. The established panels of MAbs from YFV-E protein (4A1, 4C9, 5H2, and 4H10) and MAbs from YFV-17D (3F4, 8H3, 5B6, and 3B6) were selected based on three salient features: (i) reactivity with YFV antigens by indirect ELISA; (ii) reactivity with recombinant YFV-E protein in Western blotting under reduced conditions; and (iii) reactivity with native YF-17D as shown by immunofluorescence assay. All eight of the MAbs reacted with YFV antigens in different configurations. The generation of MAbs against YFV offers important tools for YFV research, especially for diagnosis and surveillance.

Epitope mapping of the eight MAbs showed overall binding of five MAbs to domain I and II regions of YFV envelope proteins. However, three MAbs did not show strong reactivity to any of the six fragments. It is not known why these three MAbs failed to bind to all six of the fragments and yet showed strong fluorescence with YF virus-infected cells. It is possible that they may have an affinity for conformational epitopes of the virus. Binding epitopes for five MAbs were mapped to fragment DoIR1 (amino acid positions 1 to 51) and fragment DoIIR1 (amino acid positions 52 to 135) of YFV envelope proteins. The availability of such panels of MAbs represents important resources that can be utilized to generate several combinations that recognize different epitopes of the virus to achieve high levels of specificity and sensitivity. This is especially important for developing surveillance tools for analysis of antigenic variability or mutations.

The observation that our MAbs reacted with wild-type YFV strains Baringo 1 and Baringo 2 (8) with no cross-reactivity to DENV and JEV indicated strong specificity, a feature extremely important in developing diagnostic tests for YF. We investigated the cross-reactivity of our MAbs to related flaviviruses such as dengue and Japanese encephalitis viruses by two methods: indirect ELISA and IFA. This knowledge is very important given the reported cross-reactivity of many serological assays in regions where infections by several flaviviruses are endemic (43, 44). We did not find any cross-reactivity of the MAbs to DENV and JEV. Cross-reactivity was checked using DENV and JEV, and the results indicated that the established MAbs strongly reacted with YFV and could be applied in the development of diagnostic tests for YF. However, further analysis designed to determine cross-reactivity with other flaviviruses would provide additional information.

The generated MAb exhibited less neutralization activity against wild-type YF virus than against vaccine virus 17D. A number of factors have been reported to determine antibody neutralization activity for flaviviruses (45–47). The eight MAbs might be binding to epitopes that play no role in neutralization of the virus. These results were not unexpected, especially in the experiments using recombinant antigens generated from E. coli for immunization.

The MAbs developed in this study can be used in complementarity with the existing molecular tests for surveillance, monitoring, and early detection of YFV in countries where YF is endemic. Furthermore, these MAbs can be applied in the development of affordable, easy-to-use diagnostic tests in order to improve access to YF testing, especially at local health care facilities. For instance, the antigen detection ELISA evaluated in this study was very sensitive and showed the ability to detect YF vaccine virus 17D at a titer of 1 × 10^3^ FFU/ml. The MAbs against YFV have the potential of application in public health programs such as surveillance of YFV in mosquitoes. It is also conceivable that this assay can be optimized further to detect viral antigen in patient serum during the acute phase of disease, often characterized by high viremia.

Current WHO recommendations for laboratory confirmation of YFV entail testing for specific IgM antibodies and/or a ≥4-fold increase in the specific serum IgG level when other flaviviruses are ruled out (48). Among the many techniques developed for early diagnosis of YF, IgM capture ELISA is still a standard serological test. In this study, we evaluated the applicability of our MAbs in IgM capture ELISA. Remarkably, our IgM capture ELISA correctly identified all YFV IgM-positive patient and vaccinee samples. The results of analyses of dengue virus and JEV samples were negative. Furthermore, the sensitivity of the IgM capture ELISA was verified using serially diluted serum samples. Both patient and vaccinee serum samples were positive at a dilution of 1:800. These findings indicated high sensitivity of the IgM capture ELISA, as it could be used even with much-diluted patient samples, thus reducing the amount of serum needed for diagnosis. Even though the sample size analyzed was small due to the limitations with respect to accessing more patient samples for assay validation, these results demonstrated the application of MAbs for YF diagnosis. It is feasible that MAbs could be utilized in the development of additional YF diagnostic tests and could be made available at local health care facilities. This would ultimately facilitate prompt detection of infection and implementation of prevention and control measures during outbreaks.

In summary, this work highlighted the generation of YFV MAbs and the potential of using MAbs in the development of various diagnostic tests that are affordable and of high sensitivity and specificity. Despite the fact that molecular diagnostic methods are very sensitive and specific, they are most effective only at the acute phase of illness. In many African countries where YF is endemic, testing for arboviruses is often not routinely done. Clinical diagnosis of YF is also complicated by the occurrence of symptomatically similar endemic febrile illnesses. This leads to delays in detection and confirmation of YF, as patients tend to first try other treatment options. At the hospital level, the patients are first treated for other fever-causing illnesses before arbovirus infection is suspected. At such times, the viremic phase of YF will have passed and antibody levels will be on the rise, making serological diagnostic tests such as IgM capture ELISA the most practical and applicable tests for YF in Africa.

## Supplementary Material

Supplemental material
