# Supplementary material for: Development and Characterization of Monoclonal Antibodies to Yellow Fever Virus and Application in Antigen Detection and IgM Capture Enzyme-Linked Immunosorbent Assay
Source: Clin Vaccine Immunol. 2016 Aug 5;23(8):689–97. doi: 10.1128/CVI.00209-16 (PMC4979174; doi:10.1128/CVI.00209-16)
Supplement: Supplemental material [file supp_23_8_689__index.html]

Supplemental material 

# Development and Characterization of Monoclonal Antibodies to Yellow Fever Virus and Application in Antigen Detection and IgM Capture Enzyme-Linked Immunosorbent Assay

## Supplemental material

- Supplemental file 1 -

  Fig. S1. Schematic representation of YFV genome and subdivision of the polyprotein. Table S1. Envelope protein fragments showing corresponding nucleotide positions of the coding region of the whole genome, positions of the amino acids, specific primers for PCR amplification, and amino acid sequence of the protein fragment.

  PDF, 417K
