# Supplementary material for: Development and Characterization of Monoclonal Antibodies to Yellow Fever Virus and Application in Antigen Detection and IgM Capture Enzyme-Linked Immunosorbent Assay
Source: Clin Vaccine Immunol. 2016 Aug 5;23(8):689–97. doi: 10.1128/CVI.00209-16 (PMC4979174; doi:10.1128/CVI.00209-16)
Supplement: Supplemental material [file CVI.00209-16_zcd999095386so1.pdf]

Supplemental Material

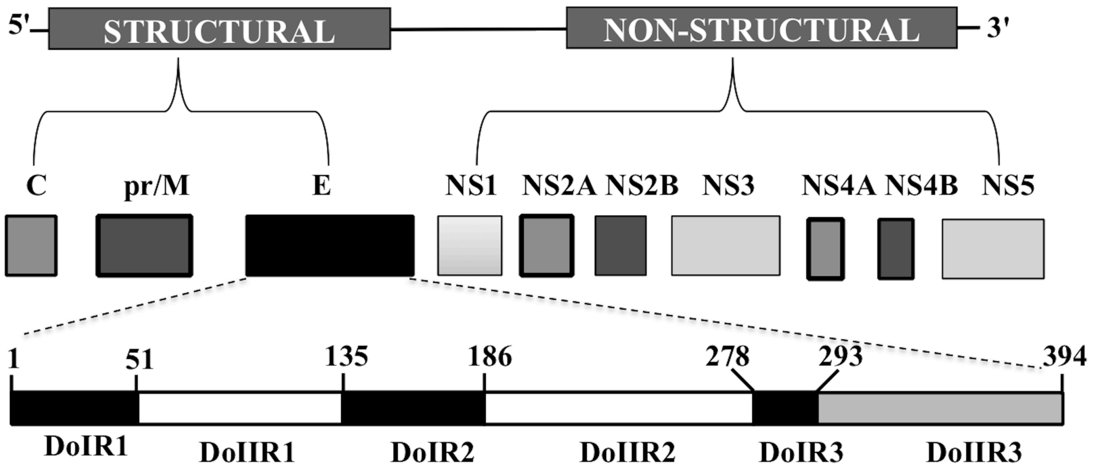

Figure S1. Schematic representation of YFV genome and sub-division of the polyprotein. The YFV E protein was sub-divided into six fragments following the segments derived from DENV-2 soluble E-protein ectodomain crystal structure. Individual fragments were cloned into pMAL-c5X plasmid vector for *E. coli* expression and purification as MBP-fusion proteins for use in epitope mapping. The six fragments were designated as DoIR1, DoIR2, DoIR3, DoIIR1, DoIIR2 and DoIIR3. The corresponding amino acid position for each fragment in the YFV protein is indicated.

Table S1. Envelope protein fragments showing corresponding nucleotide positions of the coding region of the whole genome, the positions of the amino acids, specific primers for PCR amplification and the amino acid sequence of the protein fragment.

Fragment designation	Nucleotide Position	Amino acid position[a]	Primer pairs	Amino acid sequence
**DoIR1**	973-1125	1-51	Forward 5'-atggatcctcagctcactgcattggaa -3'<br>Reverse 5'-ataagcttttaatcaatggctactgtctctagtg -3'	AHCIGITDRDFIEGVHGGTWVSATLEQD<br>KCVTVMAPDKPSLDISLETVAID
**DoIR2**	1381-1534	136-186	Forward 5'-gcggatccgtatgtcatcagagcacaat-3'<br>Reverse 5'-ataagcttttaccgcagtttgcacctggcat-3'	QYVIRAQLHVGAKQENWNTDIKTLKFD<br>ALSGSQEVEFIGYGKATLECQVQTA
**DoIR3**	1815-1845	279-293	Forward 5'-gcggatcctcttgcagagtgaaattgtcag-3'<br>Reverse 5'-gcaagcttttacattttgtcagtgcatatt-3'	SCRVKLSALTLKGTSYK
**DoIIR1**	1126-1380	52-135	Forward 5'-gtggatccagacctgctgaggtgaggaa-3'<br>Reverse 5'-ataagcttttatgaattttggtctgatcaacctcaaacaa-3'	RPAEVRKVCYNAVLTHVKINDKCPSTG<br>EAHLAEENEGDNACKRTYSDRGWGNG<br>CGLFGKGSIVACAKFTCAKSMSLFEVD<br>QTKI
**DoIIR2**	1535-1814	187-278	Forward 5'-gcggatcctggactttggtaacagttacat-3'<br>Reverse 5'-gcaagcttttaagaaacatgtccaccatgta-3'	VDFGNSYIAEMETESWIVDRQWAQDLT<br>LPWQSGSGGVWREMHHLVEFEPPHAA<br>TIRVLALGNQEGSLKTALTGAMRVTKD<br>TNDNNLYKLHGGHV
**DoIIR3**	1846-1947	294-394	Forward 5'-tcaggatcctgcattggaattact-3'<br>Reverse 5'-caacaagcttattgagcttccct -3'	ICTDKMFFVKNPTDTGHGTVVMQVKVS<br>KGAPCRIPVIVADDLTAAINKGILVTVNP<br>IASTNDDEVLIEVNPPFGDSYIIVGRGDS<br>RLTYQWHKEGSSIGKLF

[a] Position 1 represents the first amino acid of the YFV envelope protein
